# It is time to spread the message of high-quality layperson cardiopulmonary resuscitation all over the world

**DOI:** 10.1016/j.clinsp.2024.100355

**Published:** 2024-04-27

**Authors:** Naomi Kondo Nakagawa, Andrew Lockey, Maria José Carvalho Carmona, Amber Hoover, Prama Nanda, Bernd Walter Böttiger, WRAH collaborators

**Affiliations:** a Education, Assessment and Intervention in Cardiovascular Group, Faculdade de Medicina da Universidade de São Paulo, São Paulo, SP, Brazil; b Calderdale and Huddersfield NHS Trust, Halifax, United Kingdom; c Divisão de Anestesia, Hospital das Clínicas, Faculdade de Medicina da Universidade de São Paulo, São Paulo, SP, Brazil; d American Heart Association, Texas, United States of America; e European Resuscitation Council, Antwerp, Belgium; f Department of Anesthesiology and Operative Intensive Care Medicine, Faculty of Medicine, University of Cologne, Cologne, Germany

The World Restart a Heart (WRAH) initiative of the International Liaison Committee on Resuscitation is saving more lives by spreading the message of two important themes ‒ “ALL CITIZENS CAN SAVE A LIFE!” and “IT ONLY TAKES TWO HANDS TO SAVE A LIFE!” ‒ to more than 50 million citizens of the world.

The number of laypeople who undertake Cardiopulmonary Resuscitation (CPR) training has increased considerably since 2018, when the WRAH initiative was first launched,^1^ engaging 13 million people worldwide. In 2021, 200 million individuals (including social media reach) were involved.^2^ In 2022, WRAH engaged over 50 million citizens from 194 countries (Table 1). This was achieved by including over 630,000 face-to-face training, participation in witnessed live in-person demos, and attending webinars, as online readers, written readers, and television and radio audiences (Table 2). This data, which by its nature will be an approximation, has been reported and verified by national resuscitation councils from around the world. Social media campaign networks had a significant additional impact on sudden cardiac arrest and CPR awareness.

**Table 1:** World Restart a Heart collaborators, related institutions and countries.

WRAH Contributors	Institutions	Countries
Abdulmajeed Khan	Heraa General Hospital/PARC	Saudi Arabia
Aldus Smith	Resuscitation Council of Southern Africa	South Africa
Allan de Caen	University of Alberta	Canada
Amber V. Hoover	AHA/ILCOR	USA
Andrew Lockey	Resuscitation Council UK/ERC/ILCOR	UK
Antonieta Valderrama	Resuscitation Committee	Chile
	Chilean Society of Anaesthesia	
Baljit Singh	IRCF	India
Barbara Kelly	HSFC	Canada
Bassinte Ossama	Global First Aid Reference centre/IFRC	France
Bernd W. Böttiger	Medical Faculty of the University and University Hospital of Cologne/ILCOR/ERC/GRC	Germany
Farhan Bhanji	Faculty of Medicine and Health Sciences, McGill University	Canada
Federico Semeraro	Department Anaesthesia, Intensive Care and Emergence Medical System, Maggiore Hospital, Bologna/Co-Chair, ERC	Italy
Gavin Perkins	University of Warwick/Co-Chair, ILCOR/ERC	UK
George Woods	St John Ambulance	England
Jacopo Pagani	Azienda Ospedaliero-Universitaria Sant'Andrea	Italy
Jan Van Dooren	CEO, ERC	Belgium
Koen Monsieurs	Antwerp University Hospital and University of Antwerp/Chair, ERC	Belgium
Kevin Nation	New Zealand Resuscitation Council/ILCOR	New Zealand
Laurin Paris	AHA	USA
Lokesh R Edara	Western Michigan University School of Medicine/IRCF	USA
Lokesh Tiwari	All India Institute of Medical Sciences Patna /IRCF	India
Ludhmila Abrahão Hajjar	Faculty of Medicine, São Paulo University/KSLB	Brazil
Maria José Carvalho Carmona	Faculty of Medicine, São Paulo University/KSLB	Brazil
Marios Georgiou	American Medical Center Cyprus	Cyprus
Maaret Castrén	Karolinska Institute/ILCOR Honorable Secretary	Finland
Monica Sales	AHA	USA
Maureen O'Connor	San Diego Project Heart Beat	USA
Mukul Kapoor	IRCF	India
Nadine Rott	University Hospital of Cologne/GRC	Germany
Naomi Kondo Nakagawa	Faculty of Medicine, São Paulo University /KSLB	Brazil
Nilmini Wijesuriya	Resuscitation Council ‒ College of Anaesthesiologists and Intensivists of Sri Lanka	Sri Lanka
Pascal Cassan	IFRC	France
Peter Morley	University of Melbourne Royal Melbourne Hospital/ILCOR Honorable Treasurer	Australia
Prama Nanda	ERC	Belgium
Raffo Escalante-Kanashiro	Instituto Nacional de Salud del Niño/IAHF	Peru
Rakesh Garg	All India Institute of Medical Sciences New Delhi/IRCF	India
Rasesh Diwan	Indian Resuscitation Council Federation	India
Robert Neumar	University of Michigan/Co-Chair, ILCOR	USA
Shelley Parker	HSFC	Canada
Siddha SC Chakra Rao	IRCF	India
Syed Moied Ahmed	Aligarh Muslim University/IRCF	India
Swee Han Lim	Singapore General Hospital/RCA	Singapore
Teghan Mear	New Zealand Resuscitation Council/ILCOR	New Zealand
Tzong-Luen Wang	Fu-Jen Catholic University/Resuscitation Council of Asia	Taiwan
Vinay Nadkarni	University of Pennsylvania Perelman School of Medicine	USA
Zehra Al-Hilali	Arab Resuscitation Council	UAE

AHA, American Heart Association; ERC, European Resuscitation Council; GRC, German Resuscitation Council; HSFC, Heart and Stroke Foundation of Canada; IAHF, Inter-American Heart Foundation; IFRC, International Federation of Red Cross and Red Crescent Societies; ILCOR, International Liaison Committee on Resuscitation; IRCF, Indian Resuscitation Council Federation; KSLB, Kids Save Lives Brazil; PARC, Pan Arab Resuscitation Council; RCA, Resuscitation Councils of Asia; UAE, United Arab Emirates; UK, United Kingdom; USA, United States of America.

**Table 2:** World Restart a Heart: approximate numbers of people achieved in 2022.

	Face-to-face trainings	Witnessed live-in-person demos	Attending webinars	TV audience	Radio audience	Journals and magazines readers
**American Heart Association**			409,688			
**Arab Resuscitation Council**	18,322	51,415	505	10,000	5,000	
**German Resuscitation Council**	1,700			1,000,000		
**Heart & Stroke – Canada**	82,588	3,064				
**Indian Resuscitation Council Federation**	322,570	79,627	13,773	842,350	500,000	
**International Federation of Red Cross**	5,588					
**Italian Resuscitation Council**	2,000	3,000	600	5,000,000	500,000	6,183,240
**Kids Save Lives Brazil**	4,641		2,298	191,900		
**Korean Association of Cardiopulmonary Resuscitation**	731					
**New Zealand Resuscitation Council, Australian St John Ambulance**	13,000		11,628			
**Pan Arab Resuscitation Council**	22,361					
**Resuscitation Council of Southern Africa**	67,404			7,000		
**Resuscitation Council UK**	87,266	30	12,000		35,000,000	
**Singapore Resuscitation and First Aid Council**	67	2,150				
**Srilanka Resuscitation Council**	5,287		456	84,137		
**TOTAL**	**633,525**	**139,286**	**450,948**	**7,135,387**	**36,005,000**	**6,183,240**

Initiatives that combine awareness, hands-on CPR training, and the use of an external automated defibrillator may optimize bystander CPR timing, performance, and the overall quality of CPR.^3^ The target groups for face-to-face or remote CPR training are diverse and include all age groups and people from many different professional backgrounds. With specific regard to the training of children, adjustments should be made according to age range because of different physical abilities and cognitive aspects. For instance, the main objective for young children (4 years and older) would be recognition of a situation where the person does not respond and thereafter calls for help. They should know the emergency telephone number and give essential information.^4^ For children aged above 10‒12 years old, high-quality CPR and the use of a defibrillator can be achieved.^4^

High-quality bystander CPR in out-of-hospital cardiac arrest may significantly reduce the risks of brain damage, nursing home admission, or death of all causes.^5^ In this context, every year WRAH plays a relevant role as an annual awareness campaign worldwide of sudden cardiac arrest and CPR. And now, by reaching more citizens of the world, WRAH thus aims to save more lives with high-quality layperson CPR.

## Declaration of competing interest

The authors declare no conflicts of interest.
